# Isolated Superior Mesenteric Artery Dissection After Cesarean Delivery: A Case Report

**DOI:** 10.7759/cureus.113212

**Published:** 2026-07-23

**Authors:** Nobuhiko Suzuki, Naoyuki Iwahashi, Tomoko Noguchi, Sawako Minami, Kazuhiko Ino

**Affiliations:** 1 Department of Obstetrics and Gynecology, Wakayama Medical University, Wakayama, JPN

**Keywords:** acute abdominal pain, cesarean delivery, computed tomography, conservative management, hemoperitoneum, postpartum period, superior mesenteric artery dissection, visceral artery dissection

## Abstract

Isolated superior mesenteric artery dissection (ISMAD) is an uncommon vascular disorder that may present with acute abdominal pain and is most frequently reported in middle-aged men with cardiovascular risk factors. Its occurrence during the postpartum period is extremely rare, and diagnosis after cesarean delivery can be challenging because abdominal pain, anemia, abdominal distension, and hemoperitoneum may initially be attributed to postoperative bleeding or other obstetric and surgical complications.

We report the case of a 43-year-old woman, gravida 2 para 1, who developed sudden upper abdominal pain on postoperative day 4 after an uncomplicated cesarean delivery. She had no relevant medical or family history. On presentation, her blood pressure was 168/71 mmHg, and physical examination revealed mild abdominal distension and epigastric tenderness without peritoneal signs. Computed tomography (CT) revealed pelvic hemoperitoneum, and contrast-enhanced CT demonstrated an intimal flap in the superior mesenteric artery, consistent with ISMAD. There was no contrast extravasation, the distal superior mesenteric artery branches were patent, and no radiologic findings suggested bowel ischemia. Because active arterial bleeding, intestinal ischemia, and arterial rupture were absent, conservative management with strict blood pressure control, close observation, serial clinical assessment, and blood transfusion was selected. Her symptoms improved, and she was discharged without complications. Follow-up contrast-enhanced CT at 12 months showed that the dissection had become less conspicuous, and she remained recurrence-free.

This case highlights that ISMAD should be considered in postpartum women with atypical, severe, persistent, or disproportionate abdominal pain, even when postoperative bleeding appears to be a plausible explanation. Early contrast-enhanced CT is essential for distinguishing postoperative hemorrhage from visceral arterial dissection, evaluating bowel ischemia, and guiding appropriate management. To our knowledge, this is the first reported case of ISMAD after cesarean delivery.

## Introduction

Isolated superior mesenteric artery dissection (ISMAD) is an uncommon but important vascular cause of acute abdominal pain. It is defined as dissection confined to the superior mesenteric artery without concomitant aortic dissection. The superior mesenteric artery supplies a substantial portion of the small intestine and proximal colon. In ISMAD, blood entering through an intimal tear creates a false lumen that may compress the true lumen and impair mesenteric perfusion, potentially resulting in bowel ischemia or, rarely, arterial rupture [1,2]. Clinical manifestations vary from incidental detection or mild abdominal discomfort to severe pain and intestinal ischemia; therefore, diagnosis depends heavily on contrast-enhanced computed tomography (CT). Reported cases predominantly involve middle-aged men, and commonly described risk factors include hypertension, smoking, and other vascular risk factors [1-3].

The optimal management of ISMAD remains individualized. Conservative treatment is commonly selected for hemodynamically stable patients when there is no evidence of bowel ischemia, arterial rupture, or progressive vascular compromise. In contrast, endovascular or surgical intervention may be required when persistent or worsening symptoms, intestinal ischemia, arterial rupture, aneurysmal dilatation, or compromised distal perfusion is present [1,4-6]. Thus, the initial imaging assessment should not only establish the diagnosis but also stratify the risk of complications.

ISMAD during the postpartum period is extremely rare. This setting is diagnostically challenging because abdominal pain after cesarean delivery is more commonly attributed to postoperative pain, ileus, infection, uterine involution, intra-abdominal bleeding, or other obstetric and surgical complications. Only one case of ISMAD after childbirth has been reported previously [7].

We report, to our knowledge, the first case of ISMAD after cesarean delivery. The case emphasizes the need to consider visceral arterial dissection when postpartum abdominal pain is atypical, severe, persistent, or disproportionate to the expected postoperative course.

## Case presentation

A 43-year-old woman, gravida 2 para 1, underwent cesarean delivery at the referring hospital. She had no relevant medical history, no known connective tissue disorder, and no family history of vascular disease. Although the procedure was reported to have been uncomplicated, detailed operative information regarding intra-abdominal adhesions, peritoneal or mesenteric findings, surgical manipulation, and abdominal closure techniques was not available. On postoperative day 4, she developed sudden upper abdominal pain. She had not experienced similar abdominal symptoms before delivery.

On presentation, her blood pressure was 168/71 mmHg. Physical examination revealed mild abdominal distension and epigastric tenderness without rebound tenderness, guarding, or other peritoneal signs. Laboratory testing showed leukocytosis with a white blood cell count of 12,300/µL, anemia with a hemoglobin level of 7.8 g/dL, a mildly elevated C-reactive protein level of 1.78 mg/dL, and fibrinogen of 403 mg/dL. The anemia and pelvic hemoperitoneum initially raised concern for postoperative bleeding. In contrast, the leukocytosis and mildly elevated C-reactive protein level were considered nonspecific findings in the early postoperative and postpartum setting and were not, by themselves, considered evidence of bowel ischemia or severe intra-abdominal infection.

**Figure 1:**
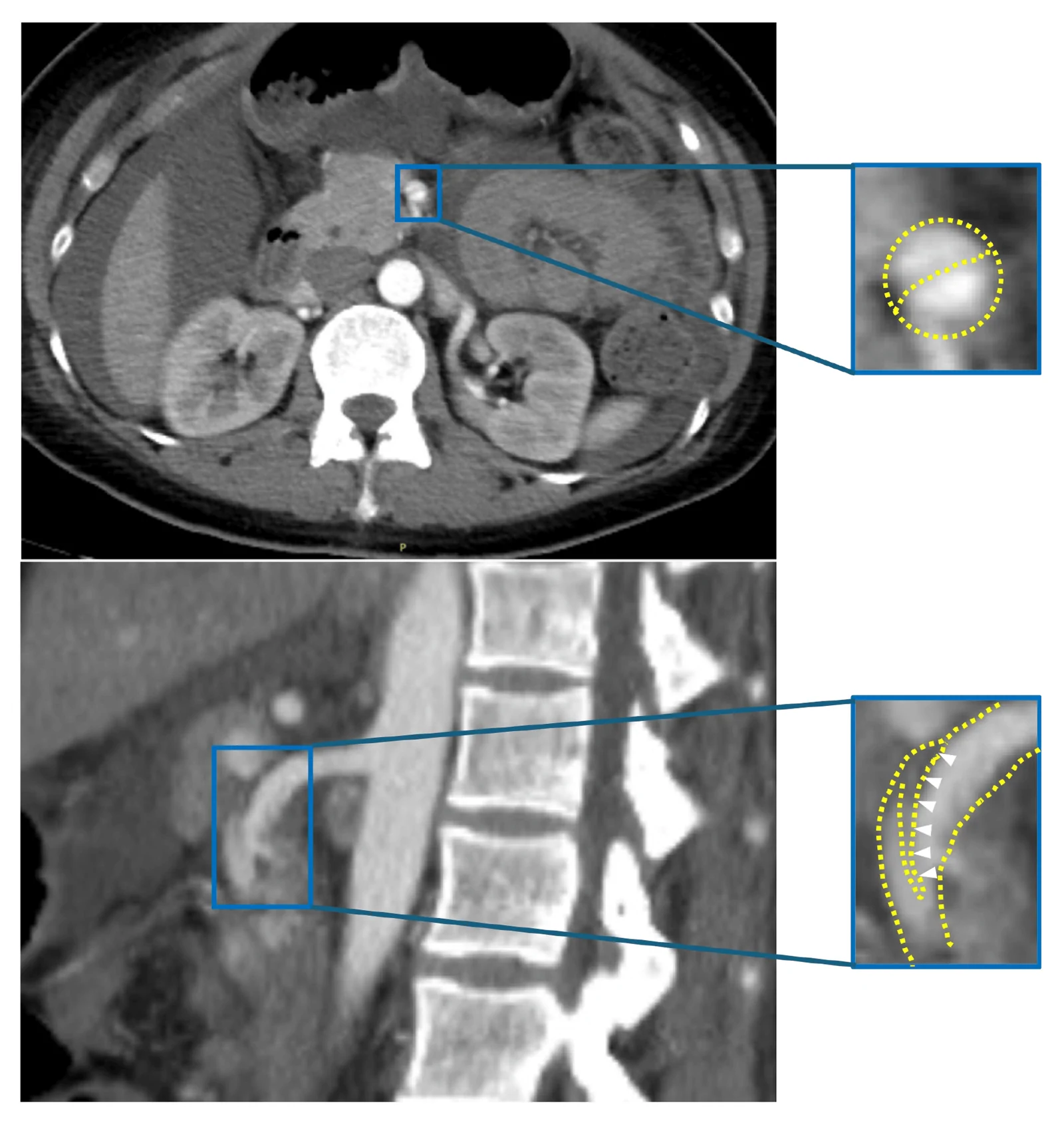
Contrast-enhanced computed tomography showing isolated superior mesenteric artery dissection after cesarean delivery. An intimal flap is visible within the superior mesenteric artery (arrowhead), consistent with arterial dissection. The distal branches remain patent, and there is no contrast extravasation or radiologic evidence of bowel ischemia.

CT revealed pelvic hemoperitoneum. Contrast-enhanced CT demonstrated an intimal flap in the superior mesenteric artery (Figure 1), consistent with ISMAD. No contrast extravasation was observed. The distal branches of the superior mesenteric artery were patent, and there were no radiologic findings suggestive of intestinal ischemia, including bowel wall thickening, reduced bowel wall enhancement, pneumatosis intestinalis, or portal venous gas. The principal diagnostic clues were the sudden onset and upper abdominal location of the pain, which were not fully explained by the pelvic hemoperitoneum, and the identification of an intimal flap in the superior mesenteric artery on contrast-enhanced CT. The patency of the distal branches and absence of radiologic signs of bowel ischemia were also important in guiding conservative management.

Because active arterial bleeding was absent, the pelvic hemoperitoneum was considered more likely to have originated from the cesarean section site than from the arterial dissection. The absence of arterial rupture, distal branch occlusion, and bowel ischemia supported nonoperative management. Conservative treatment with strict blood pressure control, close observation, repeated clinical assessment, and blood transfusion was selected. Her abdominal pain gradually improved, and no signs of intestinal ischemia or hemodynamic instability developed during hospitalization.

She was discharged without complications. Follow-up contrast-enhanced CT performed 12 months later showed that the dissection had become less conspicuous. The patient remained recurrence-free and had no subsequent abdominal symptoms.

## Discussion

This case has two main clinical implications. First, ISMAD can occur in the early postpartum period after cesarean delivery, even in a woman without a relevant medical or family history or evidence of connective tissue disease. ISMAD predominantly affects middle-aged men, with a systematic review reporting a mean age of 55.7 years and a male predominance of 80.6% [2]. In contrast, our patient was a young woman who developed ISMAD during the early postpartum period following cesarean delivery. Although a previous case of ISMAD occurring shortly after childbirth has been reported [7], to the best of our knowledge, the present case is the first reported occurrence of ISMAD following cesarean delivery. The patient’s age, sex, obstetric status, and temporal association with cesarean delivery therefore distinguish this case from the typical population affected by ISMAD and further highlight its rarity. Second, the diagnosis may be easily overlooked because the clinical picture can mimic more common postoperative complications. In the present case, anemia, abdominal distension, and pelvic hemoperitoneum made postoperative bleeding a plausible initial consideration. However, the sudden onset of upper abdominal pain and the contrast-enhanced CT findings revealed a visceral arterial dissection that required a different diagnostic and management approach.

ISMAD is increasingly recognized because of the widespread use of high-resolution CT. Nevertheless, it remains uncommon and is reported predominantly in middle-aged men. Systematic reviews have shown that many patients can be managed conservatively, particularly when intestinal ischemia and arterial rupture are absent [1-3]. The postpartum setting is markedly different from the typical patient population. Pregnancy and the postpartum period are associated with dynamic hemodynamic, hormonal, and coagulation changes, but the exact mechanism by which childbirth or cesarean delivery might contribute to ISMAD remains uncertain. Therefore, any causal relationship should be interpreted cautiously.

The key diagnostic lesson of this case is the importance of avoiding premature diagnostic anchoring after cesarean delivery. Postpartum abdominal pain is common, and postoperative bleeding, ileus, endometritis, urinary tract complications, and gastrointestinal disorders are often considered first. However, severe, abrupt, persistent, or anatomically unusual pain should prompt evaluation for less common but potentially serious vascular conditions. Contrast-enhanced CT is particularly useful because it can simultaneously evaluate postoperative hemorrhage, active extravasation, the mesenteric vessels, bowel perfusion, and other intra-abdominal complications.

Management of ISMAD should be based on symptoms, hemodynamic status, vascular morphology, distal perfusion, and the presence or absence of intestinal ischemia. Conservative management generally includes blood pressure control, bowel rest or dietary modification when clinically needed, analgesia, close clinical observation, and follow-up imaging. Endovascular or surgical treatment is usually reserved for patients with persistent or worsening abdominal pain, bowel ischemia, arterial rupture, aneurysmal progression, or failure of conservative therapy [4-6]. In the present case, conservative treatment was considered appropriate because there was no contrast extravasation, the distal superior mesenteric artery branches were patent, and there were no imaging signs of bowel ischemia.

The postpartum case previously reported after childbirth was also managed conservatively and had no recurrence during long-term follow-up [7]. To our knowledge, the present case is the first reported case of ISMAD after cesarean delivery. This distinction is clinically relevant because cesarean delivery introduces postoperative factors that can obscure the diagnosis, particularly anemia and hemoperitoneum. The diagnosis should therefore be considered not only in spontaneous postpartum abdominal pain but also in postoperative patients when the symptoms are disproportionate or not fully explained by routine postoperative findings.

Follow-up imaging is important after conservative treatment. Morphologic improvement or remodeling of the dissected segment can occur over time, but surveillance is needed to confirm stability and detect potential complications such as progression, aneurysmal dilatation, or branch compromise [8]. In this patient, follow-up contrast-enhanced CT at 12 months showed that the lesion had become less conspicuous, supporting the appropriateness of the initial conservative strategy.

This report has several limitations. First, it describes a single patient, and the mechanism of ISMAD after cesarean delivery cannot be determined from this case alone. Second, because the cesarean delivery was performed at the referring hospital, detailed intraoperative information, including the presence of adhesions, peritoneal or mesenteric abnormalities, the extent of intra-abdominal manipulation, and the closure technique, was unavailable. Therefore, we could not assess whether any specific surgical maneuver or mechanical factor contributed to the development of ISMAD, and the temporal association with cesarean delivery should not be interpreted as evidence of causality. In addition, because postpartum ISMAD is extremely rare, the optimal duration and frequency of follow-up imaging remain uncertain. Despite these limitations, this case provides a practical message for clinicians: when postpartum abdominal pain is atypical, severe, persistent, or disproportionate to the expected postoperative course, contrast-enhanced CT can be decisive in identifying uncommon vascular causes and preventing delayed diagnosis.

## Conclusions

This single case suggests that ISMAD should be considered in postpartum patients with sudden, atypical, severe, or disproportionate abdominal pain, even when postoperative bleeding appears plausible. Contrast-enhanced CT is essential for identifying visceral arterial dissection, evaluating distal perfusion and bowel ischemia, and guiding management. Although this case does not establish a causal relationship between cesarean delivery and ISMAD, conservative treatment may be appropriate in carefully selected, hemodynamically stable patients without bowel ischemia, arterial rupture, or progressive symptoms. Further reports are needed to clarify the underlying mechanisms and optimal management of postpartum ISMAD.
